# Supplementary material for: Bacterial filamentation as a mechanism for cell-to-cell spread within an animal host
Source: Nat Commun. 2022 Feb 4;13:693. doi: 10.1038/s41467-022-28297-6 (PMC8816909; doi:10.1038/s41467-022-28297-6)
Supplement: Supplementary file 1 — Supplementary Information [file 41467_2022_28297_MOESM1_ESM.pdf]

Title: Bacterial filamentation is an in vivo mechanism for cell-to-cell spreading

Authors: Tuan D Tran[1], Munira Aman Ali[1], Davin Lee[1], Marie-Anne Félix[2], Robert J Luallen[1*]

Affiliations:

[1]Department of Biology, San Diego State University, San Diego, CA 92182, USA

[2]Institut de Biologie de l' École Normale Supérieure, Centre National de la Recherche Scientifique, INSERM, École Normale Supérieure, Paris Sciences et Lettres, Paris, France

[*]Corresponding author. Email: rluallen@sdsu.edu

SUPPLEMENTARY INFORMATION

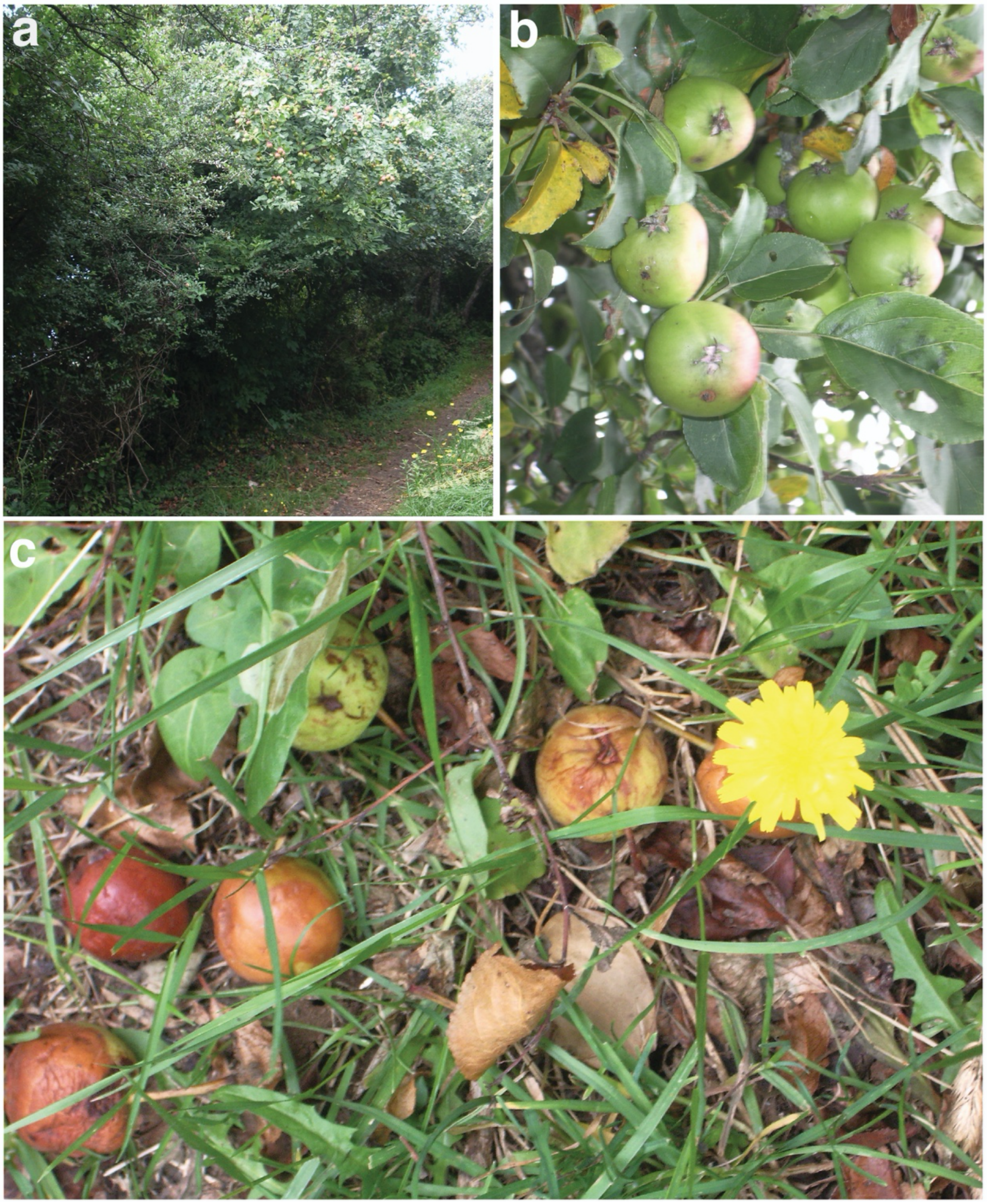

Supplementary Fig. 1**. Rotting European crab apples sampled in Kerarmel, Plouezoc'h, France. a** A wild crab apple tree with **b** ripening fruit on the stem and **c** rotting fruit below. The rotting apples were 23-25 mm in width and were taken for wild nematode sampling. *O. tipulae* strain JU1501 was isolated from these samples.

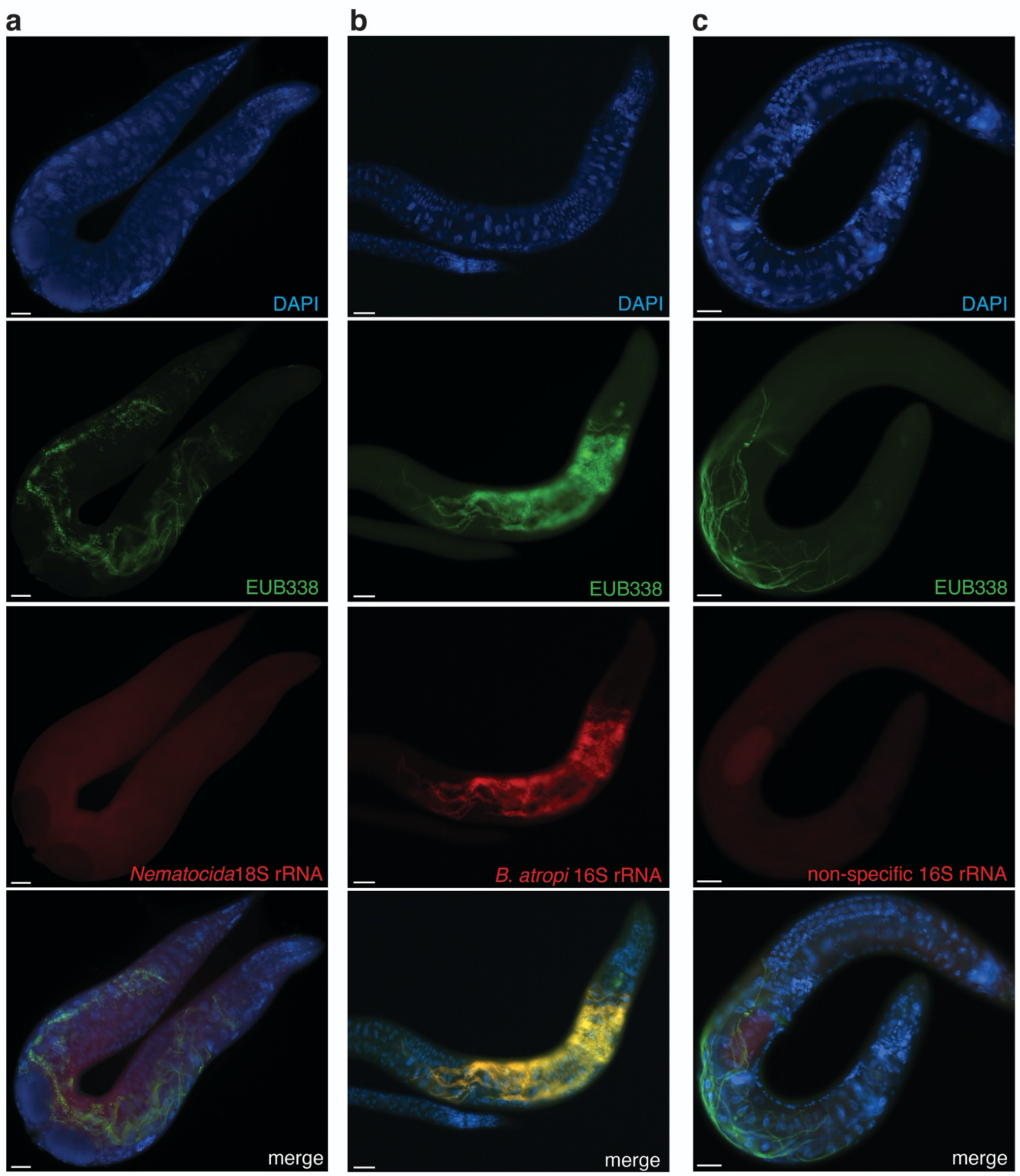

Supplementary Fig. 2. **Fluorescent micrographs of wild *O. tipulae* strain JU1501 infected with *B. atropi*.** Animals were stained by DAPI, a universal bacterial 16S rRNA probe labeled with FAM (EU338), and FISH probes labeled with CF610 specific to the small subunit of microbial rRNA, either **a** *Nematocida* 18S probes microA, microC, microE, **b** *B. atropi* 16S probe b004, or **c** Alphaproteobacteria 16S probe b002. Scale bars are 25 μm.

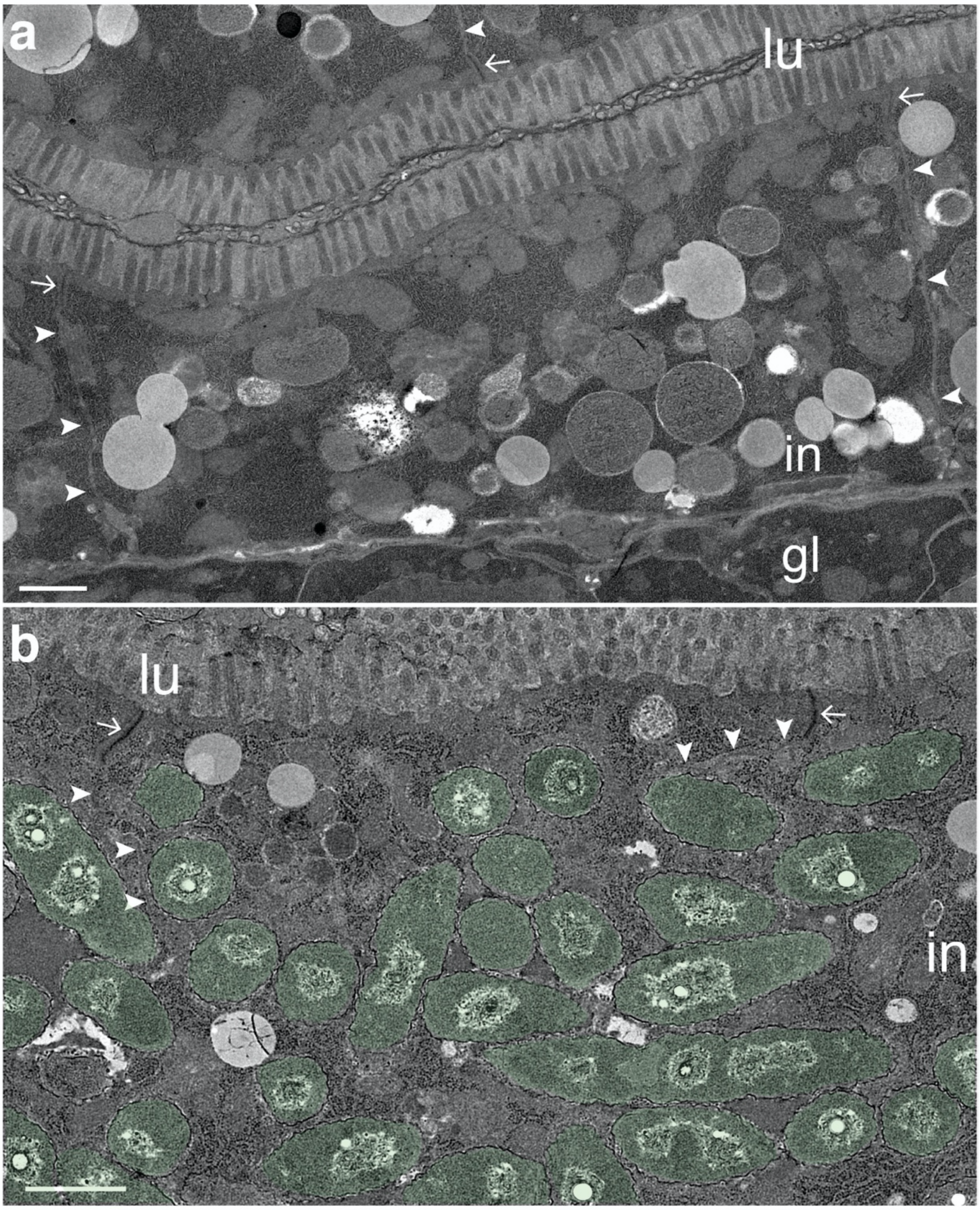

Supplementary Fig. 3. **TEM images of *B. atropi* phenotypes in *O. tipulae*. a** Uninfected and **b** infected intestine at 48 hpi showing the lumen (*lu*) with electron dense apical junctions (*arrows*) followed by the lateral intestinal membranes (*arrowheads*). The germline is indicated (*gl*). Scalebars are 1 µm.

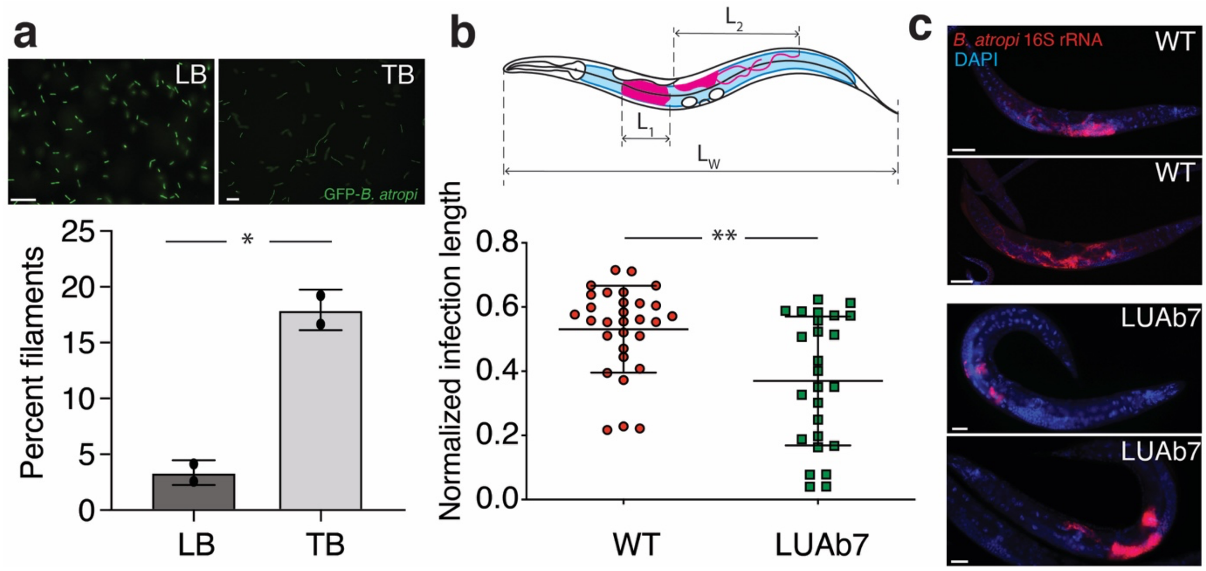

Supplementary Fig. 4. **Filamentation of *B. atropi* in TB and anterior-posterior (A-P) spreading in vivo. a** GFP-*B. atropi* was grown in LB ON and was transferred to LB or TB at 32°C for 48 hours. Bacteria were binned as filaments if greater than 4 µm in length, representing >4 bacterial cell lengths. Bar graphs show means and error bars represent standard deviation. Each point represents an independent experiment, n=2 with p=0.011 (*) by the unpaired two-tailed t test. Representative images are shown on top. Scale bars are 10 µm. **b** Animals were infected for 34 hours and stained with FISH. A schematic for describing A-P spreading (top), where the length of each contiguous infection in an animal was measures along the A-P axis (L_1, L_2,…,L_N) and summed. This total infection length was normalized to the A-P length of the animal (L_W) giving the normalized infection length (*top*). Results are from n=30 (WT), and 26 (LUAb7) examined over 2 independent experiments. Means are shown and error bars represent standard deviation, p=0.002 (**) by the Mann Whitney two-tailed t test (*bottom*). **c** Representative images from **b** are shown. Scale bars are 20 µm.

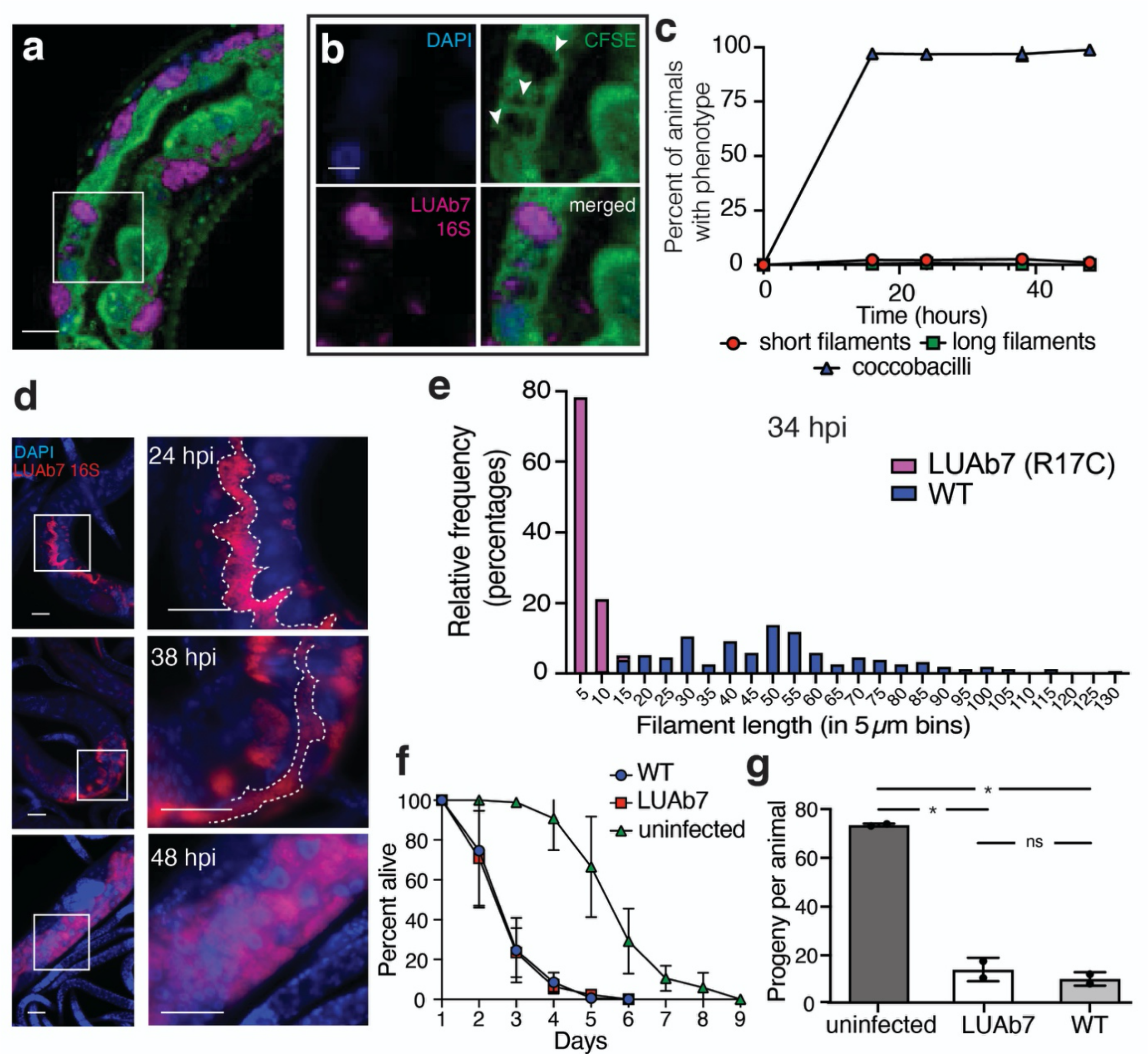

Supplementary Fig. 5. **Characterization of LUAb7 in vivo phenotypes. a** Representative confocal image of an animal infected with LUAb7. **b** Inset of region indicated by white box in **a** showing the overlap of CFSE-fluorescence clearing (arrowheads) with FISH signal from the *B. atropi*-specific 16S probe. **c** Pulse chase infection time course of two independent experiments is shown for LUAb7, n > 200 animals for each time point. **d** Representative images of phenotypes at indicated time points in **c**. Dashed lines delineate the lumen. **e** Distribution of in vivo filament lengths of LUAb7 compared to WT, n=30 animals in 2 independent experiments. **f** Life span of animals infected with either WT or LUAb7 compared to uninfected animals, n=40 animals in 2 independent experiments. Error bars represent SD. **g** Broodsize of animals infected with LUAb7 compared to WT and uninfected animals, n=40 animals in 2 independent experiments, p=0.0345 and 0.0157 (LUAb7 and WT, respectively) by unpaired, two-tailed t test with Welch's correction. Error bars represent SD. Each point represents an independent experiment. Scale bars are 5 μm in **b** and 10 μm elsewhere.

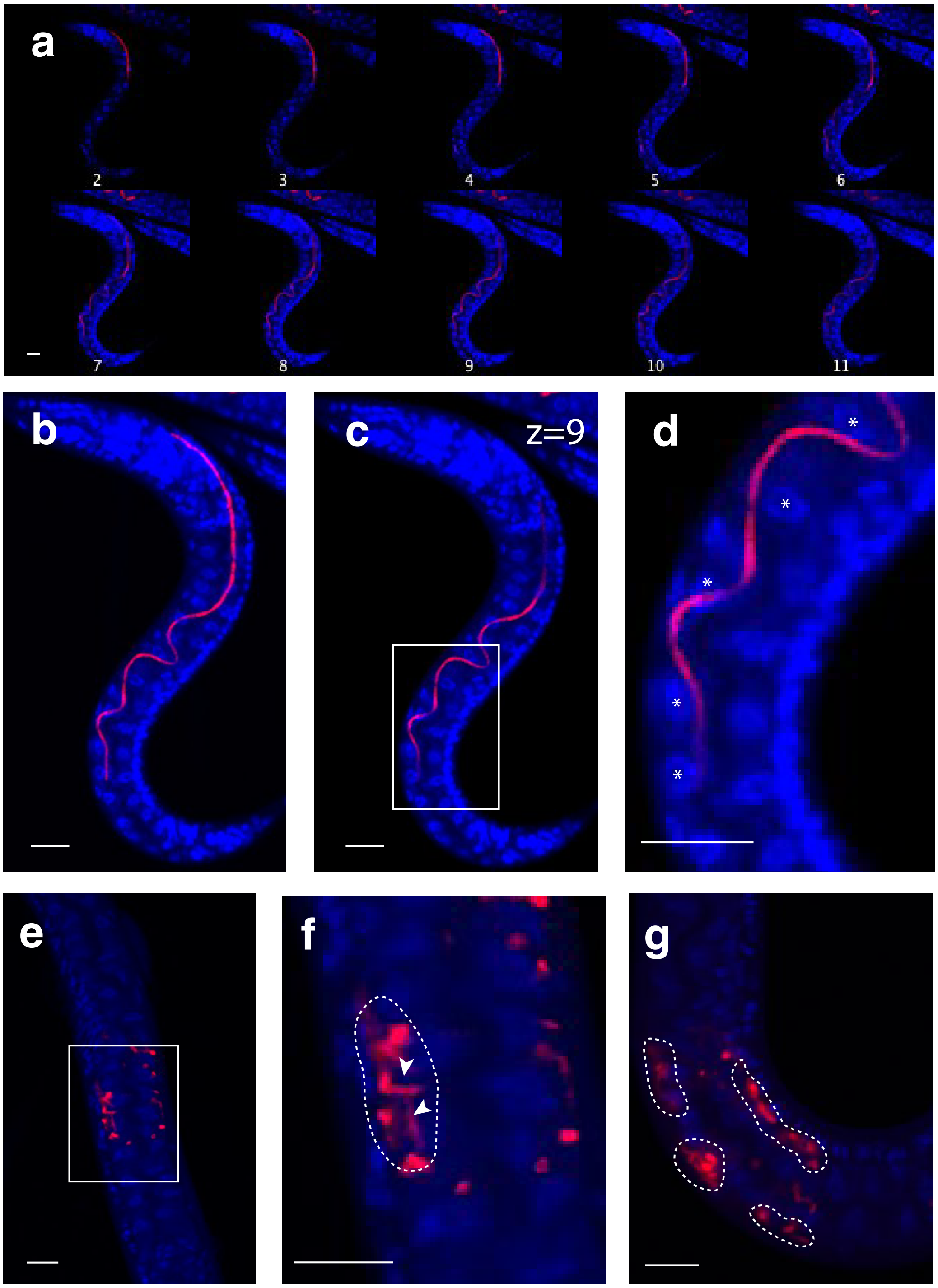

Supplementary Fig. 6. **Contiguous infection events. a** A representative montage of an WT *B. atropi* infected animal with a distinguishable "filament" infection event (numbers indicate z planes). **b** Z-projection image of the animal in **a** showing the full length of a filament. **c** Representative plane 9 showing a filament passing by multiple intestinal cells. **d** Zoomed-in region in the white box in **c** with counted intestinal nuclei indicated by asterisks. **e** An example of a LUAb7-infected animal with an infection focus. **f** Inset of region indicated in white box in **e** showing short filaments (arrowheads) closely spaced to one another and nearby coccobacilli assumed to originate from a single infection event. **g** An example of an animal with multiple infection foci (dashed line). Scale bars are 10 μm.

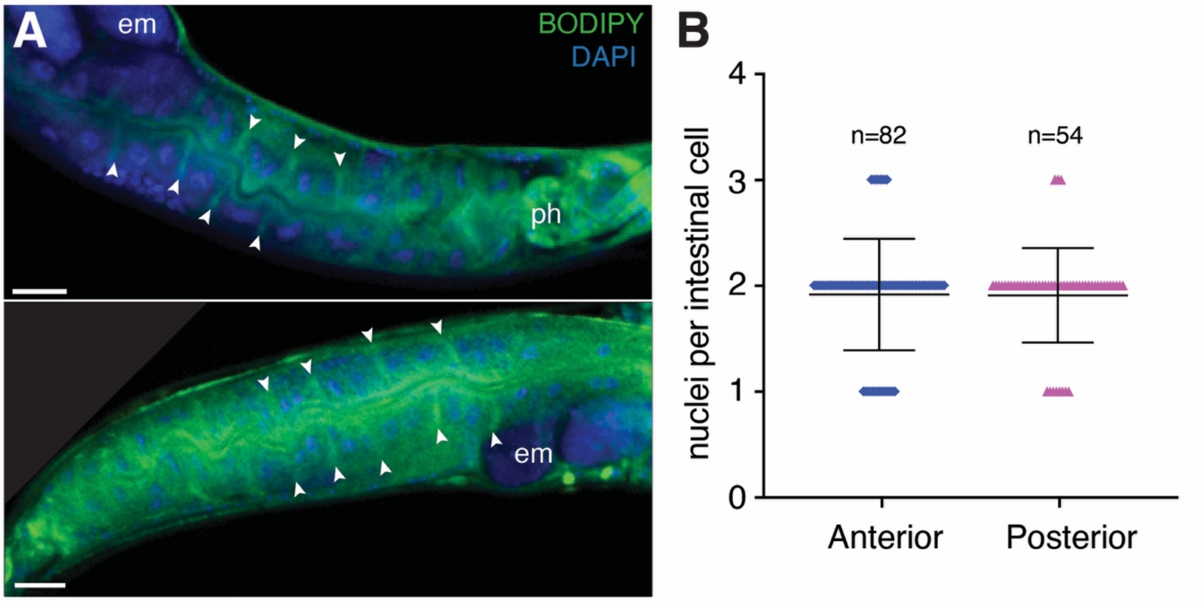

Supplementary Fig. 7. ***O. tipulae* intestinal cells contain an average of 2 nuclei at the anterior and posterior.** **a** Confocal images of *O. tipulae* animals stained with mixture of CellBrite, BODIPY-ceramide and DAPI, with lateral intestinal membranes indicated (*arrowheads*), as well as the pharynx (*ph*), and embryos (*em*). Scale bars are 20 μm. **b** The number of nuclei in intestinal cells with distinctly stained lateral membranes was counted at the anterior half and posterior half of several animals. The mean (1.9 for both) and SD are shown.

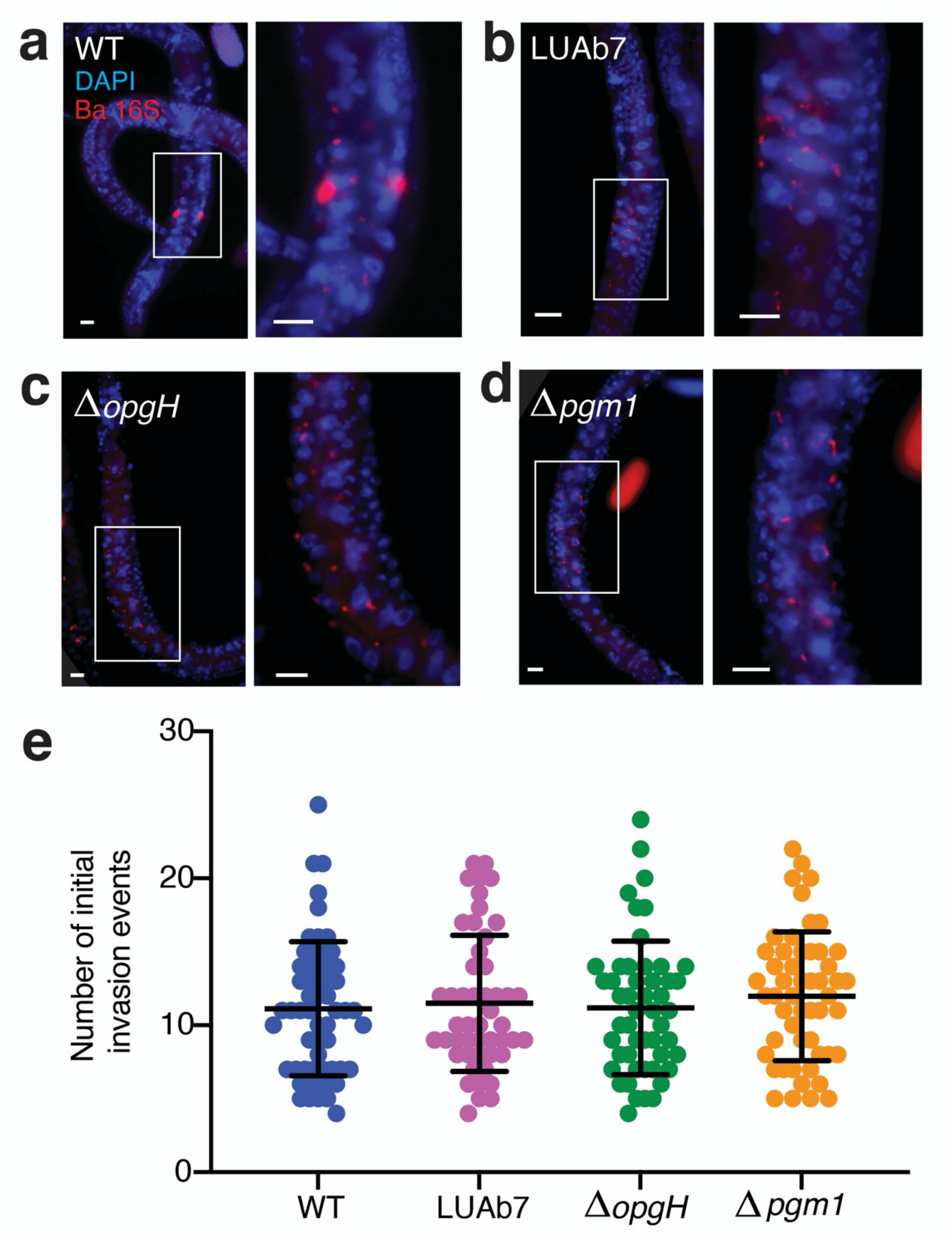

Supplementary Fig. 8. **Short pulse-chase infection results in similar initial invasion events across different strains at 16 hpi. a-d** Representative images of animals infected with WT, LUAb7, or knockout strains at 16 hpi showing similar numbers of invading bacteria. White boxes indicate regions of interest examined at higher magnification. Scale bars are 5 μm. **e** Quantification of **a-d**. Results are from n=58 (WT), 49 (LUAb7), 50 (Δ*opgH*), and 52 (Δ*pgm1*) animals examined over 2 independent experiments. Error bars represent SD.

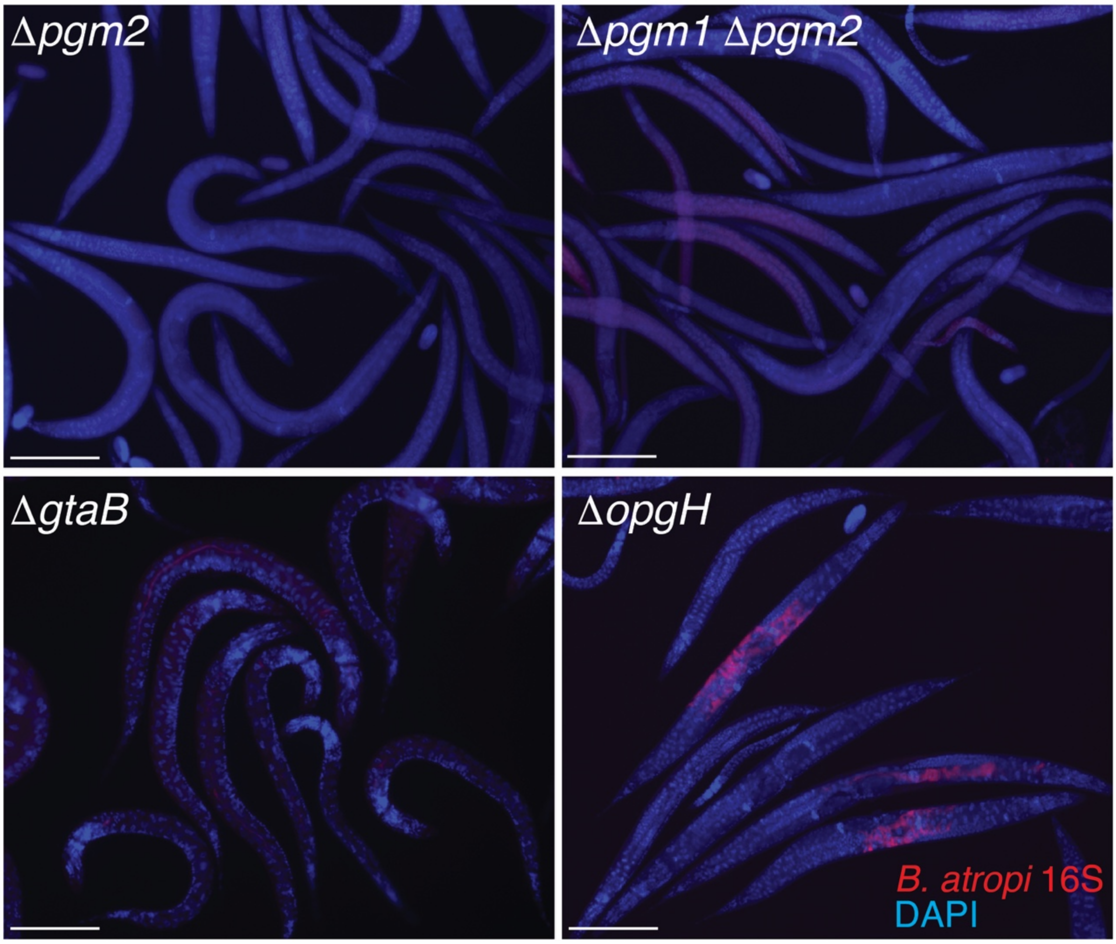

Supplementary Fig. 9. **Lack of in vivo infection in *pgm2* and *gtaB* knockout mutants.** JU1501 *O. tipulae* animals were pulse infected for 2 hours with indicated *B. atropi* strains and harvested 34 hpi for staining with 16S FISH and DAPI. Scale bars are 50 μm.

Supplementary Table 1. **Primers used for bacterial strains used in this study**

**Primer name**	**Sequence**	**Notes**
ARB1	GGCCACGCGTCGACTAGTACNNNNNNNNNNGATAT	Verify insertion site of Tn7::tdTomato in *B. atropi*
ARB2	GGCCACGCGTCGACTAGTAC	
Tn7-glmS	AATCTGGCCAAGTCGGTGAC	
gtaB_KO_Gbsn_A1	TGATGGGTTAAAAAGGATCGATCCTCTAGATCAGCTCTTGAAGCGTTGCAG	Δ*gtaB* construct
gtaB_KO_Gbsn_B1	GTGCTTACTCTTTGTCGGGTTTTCTGATAGGGCGCATTGG	
gtaB_KO_Gbsn_C1	CCAATGCGCCCTATCAGAAAACCCGACAAAGAGTAAGCAC	
gtaB_KO_Gbsn_D1	TTTTGAGACACAACGTGAATTCAAAGGGAGAGCTCTGGAAAGAGATGGGCCTG	
pgm_KO_Gbsn_A1	TGATGGGTTAAAAAGGATCGATCCTCTAGATCACTGGTGATCGCATCG	Δ*pgm1* construct
pgm_KO_Gbsn_B1	GGGGAATTTATGCAATCCATACTGCGGCTGCCGTTCTAG	
pgm_KO_Gbsn_C1	CTAGAACGGCAGCCGCAGTATGGATTGCATAAATTCCCC	
pgm_KO_Gbsn_D1	TTTTGAGACACAACGTGAATTCAAAGGGAGAGCTCATGACGTCCTACATGGAC	
algC_2_KO_Gbsn_A1	TGATGGGTTAAAAAGGATCGATCCTCTAGAATGACGAATCGCTATCAACAG	Δ*pgm2* construct
algC_2_KO_Gbsn_B1	CGTCGACAATTCCCGCATCGGATGTGAAGCTGCCGTTCTAA	
algC_2_KO_Gbsn_C1	TTAGAACGGCAGCTTCACATCCGATGCGGGAATTGTCGACG	
algC_2_KO_Gbsn_D1	TTTTGAGACACAACGTGAATTCAAAGGGAGAGCTCGCTCTGCGCGACGTTCTC	

opgH_KO_Gbsn_A1	TGATGGGTTAAAAAGGATCGATCCTCTAGAGTGTTCTCGCTTTCCTTTCGC	Δ*opgH* construct
opgH_KO_Gbsn_B1	CATCACGCCATCTGCACTTGGTAGTCCGAGACGTCATCGAG	
opgH_KO_Gbsn_C1	CTCGATGACGTCTCGGACTACCAAGTGCAGATGGCGTGATG	
opgH_KO_Gbsn_D1	TTTTGAGACACAACGTGAATTCAAAGGGAGAGCTCATGGAAAAAATCCCGATCGAC	
