# Supplementary material for: Bacterial filamentation as a mechanism for cell-to-cell spread within an animal host
Source: Nat Commun. 2022 Feb 4;13:693. doi: 10.1038/s41467-022-28297-6 (PMC8816909; doi:10.1038/s41467-022-28297-6)
Supplement: Supplementary file 3 — Reporting Summary [file 41467_2022_28297_MOESM3_ESM.pdf]

nature portfolio

Corresponding author(s): Robert Luallen, PhD

Last updated by author(s): Dec 26, 2021

Reporting Summary

Nature Portfolio wishes to improve the reproducibility of the work that we publish. This form provides structure for consistency and transparency in reporting. For further information on Nature Portfolio policies, see our Editorial Policies and the Editorial Policy Checklist.

Statistics

For all statistical analyses, confirm that the following items are present in the figure legend, table legend, main text, or Methods section.

n/a	Confirmed	
☐	☒	The exact sample size (*n*) for each experimental group/condition, given as a discrete number and unit of measurement
☐	☒	A statement on whether measurements were taken from distinct samples or whether the same sample was measured repeatedly
☐	☒	The statistical test(s) used AND whether they are one- or two-sided<br>*Only common tests should be described solely by name; describe more complex techniques in the Methods section.*
☒	☐	A description of all covariates tested
☐	☒	A description of any assumptions or corrections, such as tests of normality and adjustment for multiple comparisons
☐	☒	A full description of the statistical parameters including central tendency (e.g. means) or other basic estimates (e.g. regression coefficient) AND variation (e.g. standard deviation) or associated estimates of uncertainty (e.g. confidence intervals)
☐	☒	For null hypothesis testing, the test statistic (e.g. *F*, *t*, *r*) with confidence intervals, effect sizes, degrees of freedom and *P* value noted<br>*Give P values as exact values whenever suitable.*
☒	☐	For Bayesian analysis, information on the choice of priors and Markov chain Monte Carlo settings
☒	☐	For hierarchical and complex designs, identification of the appropriate level for tests and full reporting of outcomes
☒	☐	Estimates of effect sizes (e.g. Cohen's *d*, Pearson's *r*), indicating how they were calculated

Our web collection on statistics for biologists contains articles on many of the points above.

Software and code

Policy information about availability of computer code

Data collection	For in vitro bacterial growth curve, SoftMax Pro 6.3 software was used to collect OD600 of bacterial cultures in 96-well plate format on VersaMax Tunable Microplate Reader
Data analysis	B. atropi genome assembly was carried out with SPAdes (v3.13.0).<br>Genome annotation was conducted with prokka (v1.14.0).<br>For phylogenomics analyses, 92 up-to-date core genes alignment was carried out with UBCG (v3.0) and phylogenetic tree was made with MEGA X (v10.0.5).<br>For single nucleotide variation (SNV) calling, raw reads were processed with cutadapt (v2.3), mapped with bwa (v0.7.17-r1188), and SNVs were called with freebayes (v1.3.2-dirty).<br>Image were analyzed with Fiji software (version 2.1.0/1.53h).<br>Statistical analyses were done with GraphPad Prism9 (version 9.1.2(225)).

For manuscripts utilizing custom algorithms or software that are central to the research but not yet described in published literature, software must be made available to editors and reviewers. We strongly encourage code deposition in a community repository (e.g. GitHub). See the Nature Portfolio guidelines for submitting code & software for further information.

Data

Policy information about availability of data

All manuscripts must include a data availability statement. This statement should provide the following information, where applicable:

- Accession codes, unique identifiers, or web links for publicly available datasets
- A description of any restrictions on data availability
- For clinical datasets or third party data, please ensure that the statement adheres to our policy

This Whole Genome Shotgun project for B. atropi type strain LUAb4 has been deposited at DDBJ/ENA/GenBank under the accession JAHPZX000000000. The version described in this paper is version JAHPZX010000000. Raw reads from LUAb7 sequencing are available at Sequence Reads Archive (SRA) accession SRR14751734. All source data supporting this manuscript are provided with this manuscript.

Field-specific reporting

Please select the one below that is the best fit for your research. If you are not sure, read the appropriate sections before making your selection.

☒ Life sciences ☐ Behavioural & social sciences ☐ Ecological, evolutionary & environmental sciences

For a reference copy of the document with all sections, see nature.com/documents/nr-reporting-summary-flat.pdf

Life sciences study design

All studies must disclose on these points even when the disclosure is negative.

Sample size	A typical experiment with nematodes has hundreds of animals, and we are not limited to the number of animals or long period of life/ developmental cycles, therefore no sample-size calculation was performed before the experiments were carried out.<br><br>For lifespan and broodsize experiments, it has been reported to have a sample size of 15-20 worms per 6cm-plate. For all other experiments, 30 animals or 30 observations per replicate, amounting to 60-90 animals/observations over 2-3 replicates, were chosen based on previous publications in nematode studies (for instance, Kumsta, C. et al. Hormetic heat stress and HSF-1 induce autophagy to improve survival and proteostasis in C. elegans. Nat. Commun. 8, 14337 doi: 10.1038/ncomms14337 (2017); Kim, D. et al. A Conserved p38 MAP Kinase Pathway in Caenorhabditis elegans Innate Immunity. Science. 2002 Jul 26;297(5581):623-6).
Data exclusions	No data were excluded
Replication	Each experiment was done in 2-3 replicates. For experiments with 2 replicates, the variations between replicates were sufficiently small that it was very unlikely that a third replicate would deviate dramatically. For experiments with 2 replicates, all attempts at replication were successful. For experiments with 3 replicates, the trend holds across replicates despite experimental variations.
Randomization	In all of the experiments with image analyses, images were randomly taken at at least 3 different locations of the mounted samples. The only exception is the confocal imaging for quantification of number of intestinal nuclei as a readout for spreading capacity of the pathogen. For this experiment, we used scanning method to randomize and avoid bias toward imaging only animals with long filaments in the wild type samples.<br><br>Since nematodes are theoretically genetically identical once a strain has been established in laboratory setting, and animals are infected at the population level, randomization is not a concern for us. Besides, in all experiments, animals were harvested into a single pool, mixed, and aliquoted equally and randomly to different treatment groups, so even if there are any variations between animals unknown to us, those variations have been controlled via this procedure.
Blinding	For all in vitro assays of bacterial filamentation and cell sizes, researchers were blinded to group allocation during data collection and analysis. Additionally, in the time-course experiment where animals were binned according to phenotypes (short filaments, long filaments, or coccobacilli-filled), samples were blinded to the researcher during analysis.<br><br>For in vivo experiments, the phenotypes (long filaments vs. localized infection foci/coccobacilli, large differences in lifespan and broodsize between infected and uninfected animals) are highly distinctive and have a high penetrance which make blinding moot. We tried to avoid bias during data collection by randomly imaging different locations on prepared samples, or through canning of a well-mixed, mounted samples as described in Randomization section. These include, image analyses of normalized A-P length of infection areas and counting host intestinal nuclei as a proxy for spreading capacity of B. atropi. For these experiments, bias was controlled by randomization during imaging.

Reporting for specific materials, systems and methods

We require information from authors about some types of materials, experimental systems and methods used in many studies. Here, indicate whether each material, system or method listed is relevant to your study. If you are not sure if a list item applies to your research, read the appropriate section before selecting a response.

Materials & experimental systems

n/a	Involved in the study
☒	☐ Antibodies
☒	☐ Eukaryotic cell lines
☒	☐ Palaeontology and archaeology
☐	☒ Animals and other organisms
☒	☐ Human research participants
☒	☐ Clinical data
☒	☐ Dual use research of concern

Methods

n/a	Involved in the study
☒	☐ ChIP-seq
☒	☐ Flow cytometry
☒	☐ MRI-based neuroimaging

Animals and other organisms

Policy information about studies involving animals; ARRIVE guidelines recommended for reporting animal research

Laboratory animals	Experiments were done with laboratory hermaphrodite animals of the nematode Oscheius tipulae, strain JU1501 first isolated from rotten European crab apples sampled in Kerarmel, Plouezoc'h, France. JU1501 strain used for experiments has been maintained for about 2.5 years up to the time of this manuscript. The animals used for experiments are typically 2-4 days old (relative to nematode lifespan of about 9 days).
Wild animals	Study did not involve wild animals.
Field-collected samples	Strain JU1501 of Oscheius tipulae was first isolated from rotten European crab apples sampled in Kerarmel, Plouezoc'h, France. The first wild-caught nematodes from these samples were then established as a laboratory strain, subsequently cultured with standard nematode growth medium (NGM) agar plates and food source E. coli strain OP50-1, and maintained at 23 Celsius. At the end of experiments, animals were treated as bio-hazard materials.
Ethics oversight	No ethical approval or guidance required for invertebrate animals.

Note that full information on the approval of the study protocol must also be provided in the manuscript.
